# Effectiveness of the Sanyin Formula Plus Chemotherapy on Survival in Women With Triple-Negative Breast Cancer: A Randomized Controlled Trial

**DOI:** 10.3389/fonc.2022.850155

**Published:** 2022-05-26

**Authors:** Chunyu Wu, Chenping Sun, Guangyu Liu, Yuenong Qin, Xiaohong Xue, Xueqing Wu, Qun Wang, Jin Liu, Zhen Ye, Qiong Li, Wenchao Qu, Yi Wang, Shuai Zhang, Zhiming Shao, Sheng Liu

**Affiliations:** ^1^Department of Breast Surgery (Integrated Traditional and Western Medicine), Longhua Hospital, Shanghai University of Traditional Chinese Medicine, Shanghai, China; ^2^Department of Breast Surgery, Key Laboratory of Breast Cancer in Shanghai, Fudan University Shanghai Cancer Center, Shanghai, China; ^3^Department of Oncology, Shanghai Medical College, Fudan University, Shanghai, China; ^4^Department of Breast Surgery, Yueyang Hospital of Integrated Chinese and Western Medicine, Shanghai University of Traditional Chinese Medicine, Shanghai, China; ^5^Department of Breast, Shuguang Hospital, Shanghai University of Traditional Chinese Medicine, Shanghai, China; ^6^Department of Surgery (Thyroid and Breast Surgery), Shanghai Traditional Chinese Medicine Hospital, Shanghai University of Traditional Chinese Medicine, Shanghai, China; ^7^School of Longhua Clinical Medicine, Shanghai University of Traditional Chinese Medicine, Shanghai, China; ^8^Shanghai Cancer Center and Institutes of Biomedical Sciences, Shanghai Medical College, Fudan University, Shanghai, China; ^9^Longhua Hospital, Shanghai University of Traditional Chinese Medicine, Shanghai, China

**Keywords:** Sanyin formula, traditional Chinese medicine, triple-negative breast cancer, a randomized controlled trial, survival

## Abstract

**Purpose:**

To evaluate the efficacy of the Sanyin formula (SYF) plus conventional standard chemotherapy in operable triple-negative breast cancer (TNBC) patients, a randomized controlled trial was implemented at 5 hospitals and cancer centers in China between May 23, 2016, and October 31, 2019.

**Materials and Methods:**

Female patients aged 18 to 80 years with operable TNBC after definitive surgery were screened and enrolled. The exclusion criteria included metastatic disease, other tumors, or locally advanced disease. Patients were randomly divided into groups SYF plus conventional standard chemotherapy and placebo plus conventional standard chemotherapy at a ratio of 1:1. The primary endpoint of the investigation was disease-free survival (DFS), and secondary endpoints included overall survival (OS) and toxicity.

**Results:**

A total of 252 operable female TNBC patients were randomized to receive SYF plus conventional standard chemotherapy (N = 127) or a placebo plus conventional standard chemotherapy (N = 125). At a median follow-up of 51 months, 5-year DFS time was longer in those assigned to SYF plus conventional standard chemotherapy compared with placebo plus conventional standard chemotherapy (94.2%vs 85.5%, hazard ratio [HR] = 0.40; 95%CI, 0.17-0.97; *P* = 0.034). The absolute benefit for 5-year DFS was 8.7% in the SYF plus conventional standard chemotherapy group. No statistically significant difference was observed in OS between the two groups (*P* = 0.23). Patients with negative node status benefited more from SYF plus conventional standard chemotherapy treatment (HR = 0.21, *P*-interaction = 0.013) in accordance with the exploratory subgroup analyses of DFS.

**Conclusions:**

The results of the present study suggest that the traditional Chinese medicine SYF plus conventional chemotherapy regimens is an effective alternative adjuvant chemotherapy strategy for female operable TNBC patients.

**Clinical Trial Registration:**

https://www.chictr.org.cn/searchproj.aspx, identifier ChiCTR-IPR-16008590.

## Introduction

Triple-negative breast cancer (TNBC) is infiltrating breast cancer with negative estrogen receptor (ER), progesterone receptor (PR), and human epidermal growth factor receptor 2 (HER2) expressions, which renders it unresponsive to conventional anti-hormonal therapy and anti-HER2-targeted treatments (1). Although TNBC accounts for 15-20% of all female breast cancers, the metastatic occurrence rate of TNBC is the highest, and the overall survival rate is the lowest (2). Due to the lack of approved targeted therapies, surgery, cytotoxic chemotherapy and DNA damaging agents are the current established standard treatments for TNBC (3). Although chemotherapy can significantly improve the clinical outcome of TNBC patients, the recurrence rate is still relatively high, and TNBC tumors are usually resistant to chemotherapy agents (4–6). Therefore, considering the limited treatment options and relatively high invasiveness and recrudescence of TNBC, the development of new TNBC treatment options is crucial.

In China, traditional Chinese medicine (TCM) is widely used in the treatment of cancers (7). The progress of traditional Chinese medicine in the prevention and treatment of cancer has attracted the attention of many countries worldwide in recent years. The US National Cancer Institute Office of Cancer Complementary and Alternative Medicine has increased the incentives for international cooperation and has engaged in extension cooperation in traditional Chinese medicine and cancer research with the Cancer Institute of the China Academy of Chinese Medical Sciences and institutes at the China Academy of Sciences and Chinese Academy of Medical Sciences (8). In contrast to Western medicine, TCM has multiple targets, which gives it various advantages in the treatment of cancers. For example, all-*trans* retinoic acid (ATRA) increases the cellular uptake of arsenic trioxide (ATO) by upregulating aquaporin 9, while ATO inhibits the carcinogenic function of Pin1 by noncovalent binding with the Pin1 active site. ATRA/ATO therapy cooperatively ablates Pin1, which can block many cancer-driving pathways and, finally, inhibit the growth of cancer cells (9). Thus, ATRA plus ATO synergistic targeted therapy was recommended by NCCN as the first choice for acute promyelocytic leukemia (APL) treatment in 2014 (10). A meta-analysis suggested that TCM in combination with Western medicine has advantages over Western medicine in treating TNBC (11). These advantages include reducing the side effects of radiotherapy and chemotherapy, improving the quality of life of patients, preventing tumor metastasis and recurrence, and improving the survival rate of patients. Therefore, the therapeutic strategy of combining traditional Chinese medicine with Western medicine is a new and beneficial strategy for the clinical treatment of TNBC patients.

The Sanyin formula (SYF), which is a traditional Chinese medicine formula that is composed of 9 traditional Chinese medicines, is effective in reducing the recurrence and metastasis of TNBC patients during long-term clinical treatment in our hospital. In our previous prospective cohort study, we investigated the clinical efficacy of SYF for TNBC and found that SYF increased the 2-year disease free survival (DFS) (12). The 2-year DFS was 88.7% for the SYF plus conventional standard chemotherapy group, which was greater than that of the nonexposure control group (82.5%) (P < 0.05). The absolute benefit for the 2-year DFS was 6.2% for the SYF group. SYF reduced the disease-related recurrence and metastasis rate by 11.0% (OR = 0.89, 95% CI 0.37-0.96), with a statistically significant difference (*P* < 0.05). However, we do not know the longer-term benefits of SYF in TNBC patients because clinical data over periods of more than 2 years were not obtained. Additionally, this prospective cohort study was not a double-blind randomized controlled trial, and researcher bias may have been present. Therefore, we designed a multicenter, randomized, double-blind, placebo-controlled (RDBPC) trial to observe and verify the clinical efficacy and safety of SYF for operable TNBC patients. The randomized controlled trial (RCT) has been registered and approved in the Chinese Clinical Trial Registry (ChiCTR) (No. ChiCTR-IPR-16008590).

## Materials and Methods

### Study Design and Participants

The randomized double-blind placebo control trial was a randomized and multicenter clinical trial that was carried out in 5 cancer centers and hospitals in China (**Supplement Table 1**). The study protocol was approved by the independent institutional review committees of the participating centers and hospitals (**Supplementary Table 1**). This investigation followed the guidelines of the Consolidated Standards of Reporting Trials (CONSORT). The study was performed according to the ethical principles of the Declaration of Helsinki and the International Conference on Harmonisation Good Clinical Practice (ICH-GCP) guidelines. All patients provided written informed consent. Trial registration: Chinese Clinical Trial Registry (ChiCTR), ChiCTR-IPR-16008590. Registered 3 June 2016, https://www.chictr.org.cn/searchproj.aspx.

All patients were screened between May 23, 2016, and October 31, 2019. Female patients aged 18 to 80 years with operable, primary invasive TNBC were included in this study. The estrogen receptor (ER), progesterone receptor (PR), and human epidermal growth factor receptor 2 (HER2) statuses were identified according to our previous study (13). The sample size of the study was estimated using Power and Sample Size at http://powerandsamplesize.com/Calculators/ in a compare two proportions manner with the following formula according to a previous study (14).


$$n_{A}=kn_{B},\;\;n_{B}=\left(\frac{p_{A}\left(1-p_{A}\right)}{k}+p_{B}\left(1-p_{B}\right)\right){\left(\frac{z_{1-\alpha }+z_{1-\beta }}{p_{A}-p_{B}}\right)}^{2}$$

$$1-\text{β}=\emptyset \left(\frac{\left|p_{A}-p_{B}\right|}{\sqrt{\frac{p_{A}\left(1-p_{A}\right)}{n_{A}}+\frac{p_{B}\left(1-p_{B}\right)}{n_{B}}}}-z_{1-\alpha }\right)$$

Here, κ=n_A_/n_B_ is the matching ratio, ∅ is the standard normal distribution function, ∅^−1^ is the standard normal quantile function, α is type I error, and β is type II error, meaning 1−β is power. In this trial, κ = 1, α = 0.05, and β = 0.2. Substituting the formula, the calculated amount of each group is N = 123. Approximately 252 cases were eventually included.

### Inclusion Criteria

The inclusion criteria were as follows: (1) primary breast cancer cases after surgical treatment were clearly diagnosed as malignant epithelial tumors of the breast (breast cancer) by pathological examination, and the results of ER, PR, and Her-2 immunohistochemistry were all negative; (2) newly diagnosed patients had breast cancer before chemotherapy or within 3 months after chemotherapy, and there was no recurrence or metastasis; (3) Karnofsky score ≥ 60 points; (4) patients were female and 18-80 years old with an estimated survival time > 6 months; (5) patients had no severe organic or functional diseases and no drug or food allergies; and (6) patients were willing to accept treatment, observation, and various examinations.

### Exclusion Criteria

The exclusion criteria were as follows: (1) patients who did not meet the inclusion criteria; (2) patients for whom tumor markers continued to increase, there were undiagnosed masses in the pelvic or abdominal cavity or organs, or PET indicated recurrence and metastasis; (3) patients who had an obstruction and could not take traditional Chinese medicine; (4) women who were breastfeeding, pregnant, or about to become pregnant; (5) patients with allergies to multiple drugs; (6) patients with severe primary diseases of the cardiovascular, cerebrovascular, liver, kidney, or hematopoietic system or mental illness; and (7) subjects who participated in other drug tests.

### Randomization and Blinding

The eligible female cases after breast cancer surgery were randomly assigned to receive the Sanyin formula or placebo (one-tenth dose of SYF) at a ratio of 1:1. The randomization method was implemented by biostatisticians who had no knowledge of the data management or data analysis of this experiment from Shanghai BioGuider Medicinal Technology Co., Ltd. using the SAS 9.3 package in a central random system. After blinding, the blinder mailed one sealed copy of the blind codes to the hospital research office. The other copy was kept by the sponsor. Patients who met the inclusion criteria were randomly assigned after obtaining signed written informed consent. The random number and confirmation code information was entered into the DAS for the IWRS system after the blind coding was completed, and this information was used for the random number application and drug distribution. Data collection and management were managed and preserved by Shanghai BioGuider Medicinal Technology Co., Ltd. (Shanghai, China). All patients were randomly divided into two groups according to the above stratification.

### Procedures

The baseline characteristics of the participants were recorded before randomization. All patients received SYF or placebo based on the results of the randomization. All patients received SYF or placebo for at least 2 years. The medicine (dry powder, 2 bags) was administered after being fully dissolved in a suitable amount of hot water (approximately 50-60 ml). The oral dosage was 14 g bid. The detailed protocol was carried out according to our previous study (13). The prescription composition and preparation of SYF and placebo were as follows.

### Prescription Composition

The Sanyin formula is a traditional Chinese medicine formula that is composed of the following 9 traditional Chinese herbs: *Codonopsis pilosula* Nannf. (Chinese name: Dangshen), *Atractylodes macrocephala* Koidz. (Chinese name: Baizhu), *Poria cocos (Schw.)* Wolf. (Chinese name: Fuling), *Salviae chinensis* Herba (Chinese name: Shijianchuan), *Curcuma phaeocaulis* Valeton (Chinese name: Ezhu), *Epimedium brevicornu* Maxim. (Chinese name: Yinyanghuo), *Solanum nigrum* Linn. (Chinese name: Longkui), *Scutellariae barbatae* D. Don (Chinese name: Banzhilian), and *Prunella vulgaris* Linn. (Chinese name: Xiakucao). The total daily dose was 180 g. All Chinese herbal medicines were purchased from Shanghai Kangqiao Traditional Chinese Medicine Pieces Co., Ltd. and were identified by expert traditional Chinese pharmacists at Longhua Hospital.

### Preparation of SYF and the Placebo

SYF and the placebo were prepared by Tiangjiang Pharmaceutical Co., Ltd. The prescription composition (180 g), as specified above, was added at a ratio of 1:13 to pure water (2340 ml). Then, the mixture was heated to boiling and kept slightly boiling for 1 hour. The filtrate was obtained after filtration. The obtained residue was decocted again. The filtrates from the two extractions were combined. The filtrate was concentrated under reduced pressure to a liquid with a specific gravity of 1.10-1.12 (65 ± 5°C). Spray drying (process parameters: inlet air temperature 160°C-180°C, outlet air temperature 95°C-105°C) was used to obtain a dry extract powder. The dry extract was ground into 12-40 mesh granules. These particles were packed into aluminum foil bags that had been preprinted with corresponding labels. There was a total of 7.0 g Chinese medicine granules in each bag.

The placebo was obtained by mixing excipients with SYF at a ratio of 9:1. The excipients were composed of the following: lactose, caramel color, sunset yellow basic color, lemon yellow basic color, and a bittering agent, among other compounds. After the excipients were adjusted, they were mixed with the SYF mixture and packaged.

### Drug Packaging and Delivery

Drug packaging was carried out by staff who were unrelated to this study and worked at Shanghai BioGuider Medicinal Technology Co. Ltd. The drugs were packaged according to the treatment group that corresponded to the random number that was generated by the software. In this trial, a central random system was used to deliver drugs according to the visit period. Each subject received a completely consistent treatment regimen according to their unique random number. The package number of the drug was entered into the DAS for the IWRS system after blind coding was completed and was used for the drug distribution.

### Outcomes

The primary endpoint was DFS, and DFS events were identified in accordance with previous work (13). Briefly, the DFS events included noninvasive and invasive breast cancer recurrences, second primary noninvasive and invasive breast cancers, and second primary non-breast cancers, as well as death from any cause. Secondary endpoints included overall survival (OS) and toxicity. OS was defined as the time from random assignment to death from any cause.

### Unblinding

The unblinding date was June 28, 2021. The unblinding table was mailed to the researchers by a third-party data management company (Shanghai BioGuider Medicinal Technology Co. Ltd).

### Safety Evaluation

In order to assess drug safety or any adverse effects, patients were observed for safety metrics throughout the study. Blood, urine, stool routine, liver and kidney function, abdominal B-ultrasound, and electrocardiogram were performed every 3 months. If the investigator finds any adverse events (AEs), they should be graded according to the World Health Organization (WHO) criteria for presentation and indicators of acute and subacute toxicity (grades 0, 1, 2, 3, and 4). The correlation between toxic or side effects and drugs was carefully analyzed. In the event of serious adverse reactions, the trial will be terminated by the comprehensive decision of the subjects or investigators.

### Statistical Analysis

Data were analyzed from June 29, 2021, to April 22, 2022. For continuous and categorical factors, the Wilcoxon rank-sum test and the χ2 test were used to evaluate differences between the SYF group and placebo group. The distributions of survival outcomes were calculated by the Kaplan–Meier method. The stratified log-rank test was used to compare the survival curves of the two groups. The stratified Cox proportional hazards model was used to calculate the hazard ratios and 95% confidence intervals (95% CIs). The Cox regression analysis was carried out to analyze the possible interaction between the indicators of the subgroups and the prognosis. Kaplan–Meier curves were adopted to estimate the DFS and OS probabilities at 1, 3, and 5 years. A Z-test was employed to compare the 1-, 3-, and 5-year survival rates between the two groups. All analyses were performed with SPSS statistical software, version 19.0 (IBM Corp., Armonk, NY). The results were plotted using OriginPro statistical software, version 2021b (OriginLab Corp., Northampton, MA). A two-sided *P*-value or *P*-interaction < 0.05 was considered statistically significant according to usual practice.

## Results

### Patients Characteristics

**Figure 1:**
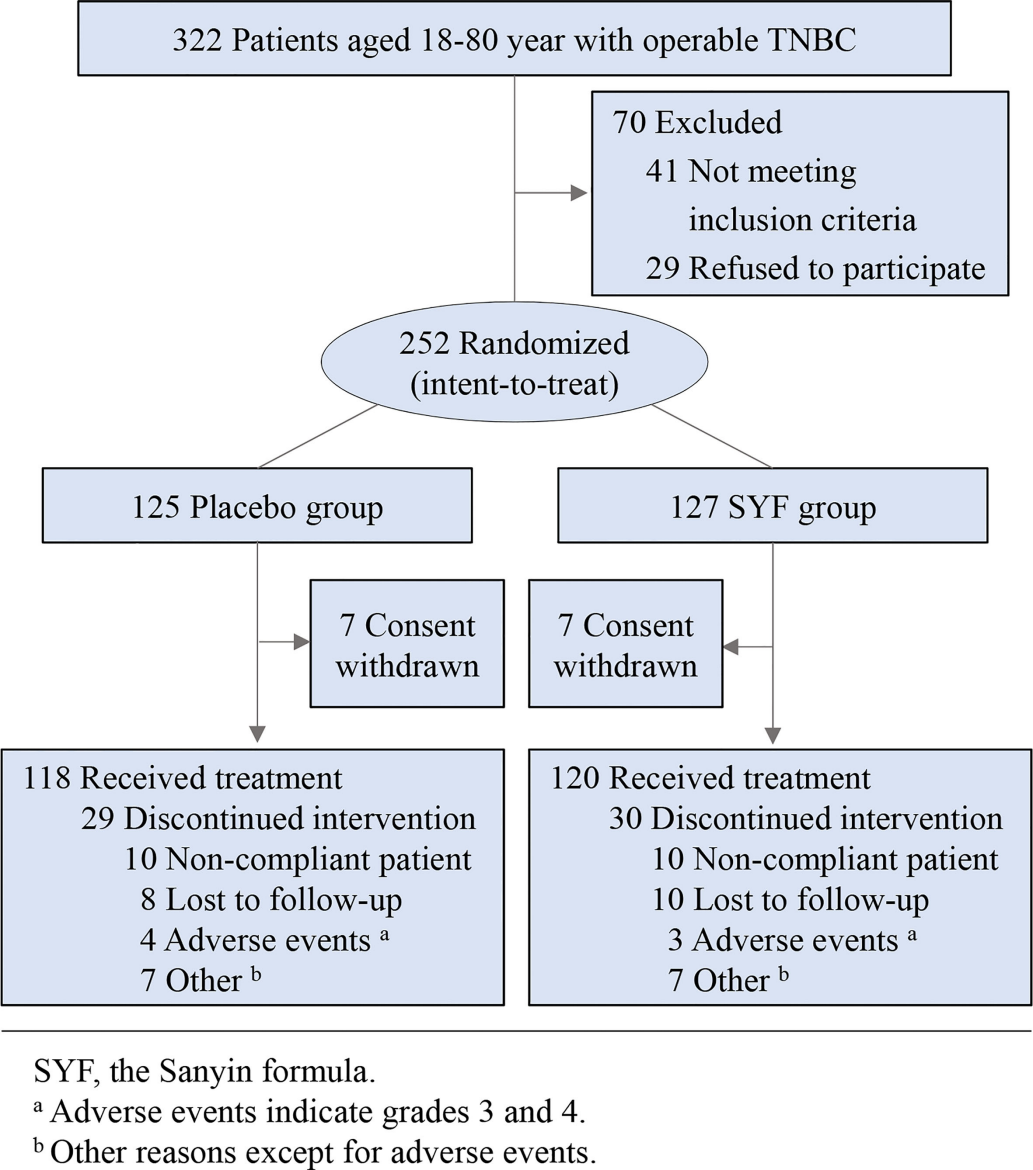
Flow diagram of the study.

A total of 322 TNBC patients were screened at 5 hospitals and cancer centers in China between May 23, 2016, and October 31, 2019. Among them, 252 patients were enrolled and randomly divided into two groups: 127 in the SYF treatment group and 125 in the placebo group (**Figure 1**). The baseline characteristics of the 252 patients who completed the investigation were well balanced between the two groups (**Table 1**). The median age of the employed patients was 51 years (interquartile range, 44-60 years) at the randomization time. The main pathological type of the enrolled patients was invasive ductal carcinoma (> 90%) (**Table 1**). Most cases were early-stage TNBC cases (node-negative rate: 62%) (**Table 1**). The chemotherapy regimen for these patients was mainly EC-P (**Supplementary Table 2**). The proportions of EC-P, EC, and CEF-T cells in the placebo group were 46%, 12%, and 11%, respectively. The rates of EC-P, EC, and CEF-T in the SYF groups were 48%, 14%, and 9%, respectively. Approximately two-thirds of the patients underwent a mastectomy to remove the tumor mass, and the others underwent BCS (**Table 1**). Tumor size, histological grade, and Ki67 proliferation index were similar between the two groups (**Table 1**). Approximately 77% of the patients completed all trials (**Table 2**).

**Table 1:** Patient baseline characteristics.

Characteristic	No. (%) of patients		
	Total (N = 252)	Placebo (N = 125)	SYF (N = 127)
**Age, median (IQR), year**	51 (44-60)	51 (44-60)	52 (45-61)
**Pathological type**			
** Invasive ductal carcinoma**	227 (90.1)	112 (89.6)	115 (90.6)
** Others**	25 (9.9)	13 (10.4)	12 (9.4)
**Age, months**			
**<50**	105 (41.7)	55 (44.0)	50 (39.4)
**≥50**	147 (58.3)	70 (56.0)	77 (60.6)
**Tumor size**			
** T1**	133 (52.8)	58 (46.4)	75 (59.1)
** T2-3**	104 (41.3)	60 (48.0)	44 (34.6)
** Unknown**	15 (5.9)	7 (5.6)	8 (6.3)
**Histological grade**			
** I-II**	58 (23.0)	28 (22.4)	30 (23.6)
** III**	171 (67.9)	87 (69.6)	84 (66.2)
** Unknown**	23 (9.1)	10 (8.0)	13 (10.2)
**Ki67 proliferation index (%)**			
**≤30**	56 (22.2)	31 (24.8)	25 (19.7)
**>30**	188 (74.6)	88 (70.4)	100 (78.7)
** Unknown**	8 (3.2)	6 (4.8)	2 (1.6)
**Node status**			
** Negative**	157 (62.3)	82 (65.6)	75 (59.1)
** Positive**	68 (27.0)	30 (24.0)	38 (29.9)
** Unknown**	27 (10.7)	13 (10.4)	14 (11.0)
**Administration**			
** Before chemotherapy**	102 (40.6)	50 (40.0)	52 (40.9)
** After chemotherapy**	150 (59.4)	75 (60.0)	75 (59.1)
**Surgery**			
** BCS**	82 (32.5)	45 (36.0)	37 (29.1)
** Mastectomy**	166 (65.9)	76 (60.8)	90 (70.9)
** Unknown**	4 (1.6)	4 (3.2)	0 (0.0)

**Table 2:** Summary of clinical trial termination.

Reasons	No. (%) of patients	
	Placebo (N = 125)	SYF (N = 127)
**Total**	29 (23.2)	30 (23.6)
**Non-compliant patient**	10 (8.0)	10 (7.9)
**Lost to follow-up**	8 (6.4)	10 (7.9)
**Withdrawal of informed consent**	7 (5.6)	7 (5.5)
**Adverse reactions**	4 (3.2)	3 (2.4)

### Efficacy

**Figure 2:**
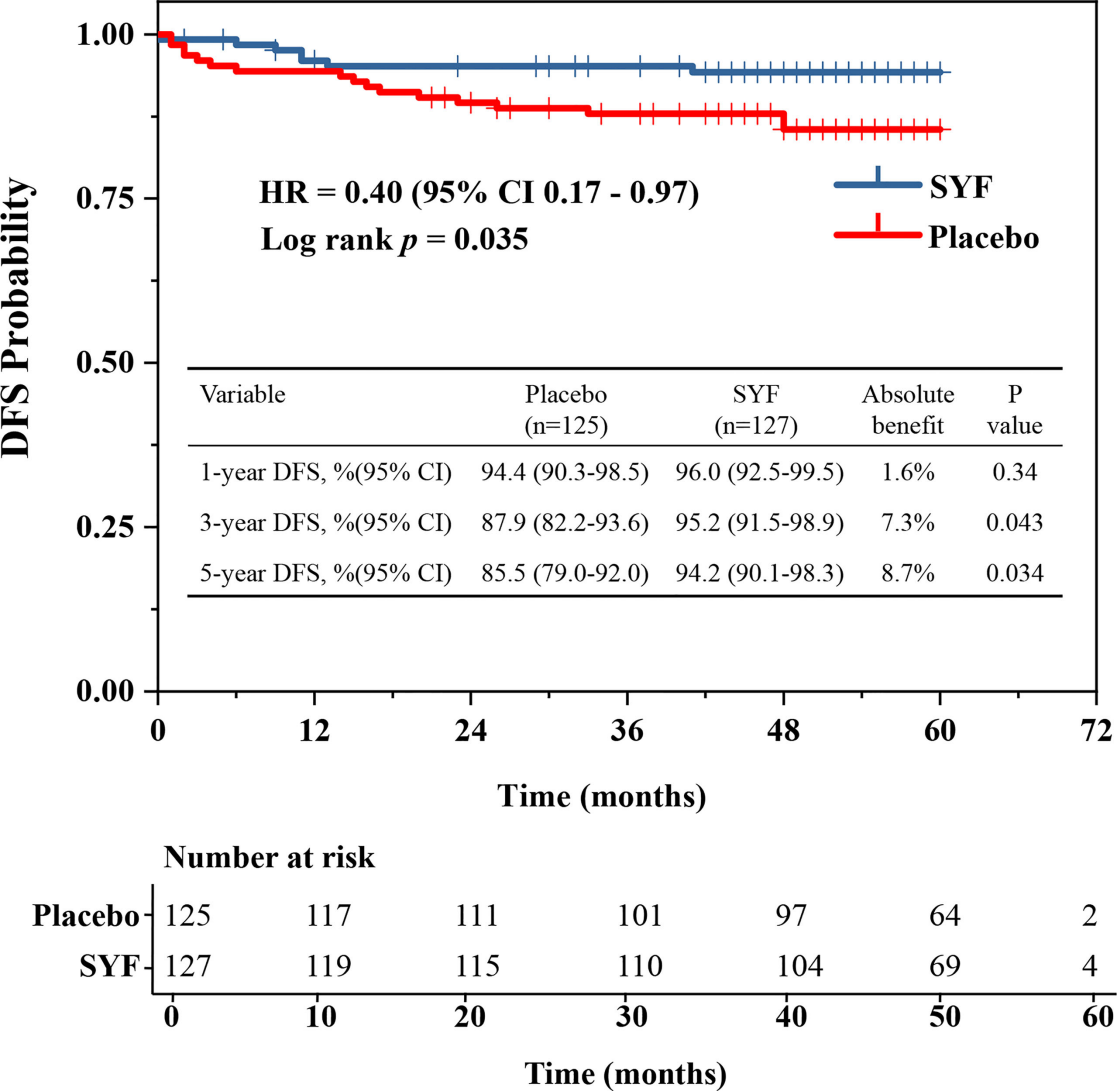
Disease-Free Survival.

All patients underwent a minimum of 42 months of follow-up. At a median follow-up of 51 months, 24 (9.5%) DFS events were observed in these female patients (**Table 3**). The results of the Kaplan-Meier curves of DFS suggested that the 5-year DFS time was longer in those assigned to SYF plus conventional standard chemotherapy compared with placebo plus conventional standard chemotherapy (94.2%vs 85.5%, *P* = 0.034) (**Figure 2**). The absolute benefit for 5-year DFS was 8.7% in the SYF plus conventional standard chemotherapy group (**Figure 2**). Only 7 DFS events among 127 TNBC cases were found in the SYF plus chemotherapy group, while 17 events were found among 125 patients in the placebo plus chemotherapy group. The HR of the SYF plus chemotherapy group was 0.40 (95% CI, 0.17-0.97), with a statistically significant difference (stratified log-rank *P* = 0.035) (**Figure 2**).

**Table 3:** First DFS event by treatment.

DFS event	No. (%) of patients	
	Placebo (N = 125)	SYF (N = 127)
**Local and regional recurrence**	6 (4.8)	1 (0.8)
**Contralateral breast tumor**	2 (1.6)	2 (1.6)
**Distant metastasis**	9 (7.2)	4 (3.1)
**Death**	5 (4.0)	2 (1.6)
**Total**	17 (13.6)	7 (5.5)

**Figure 3:**
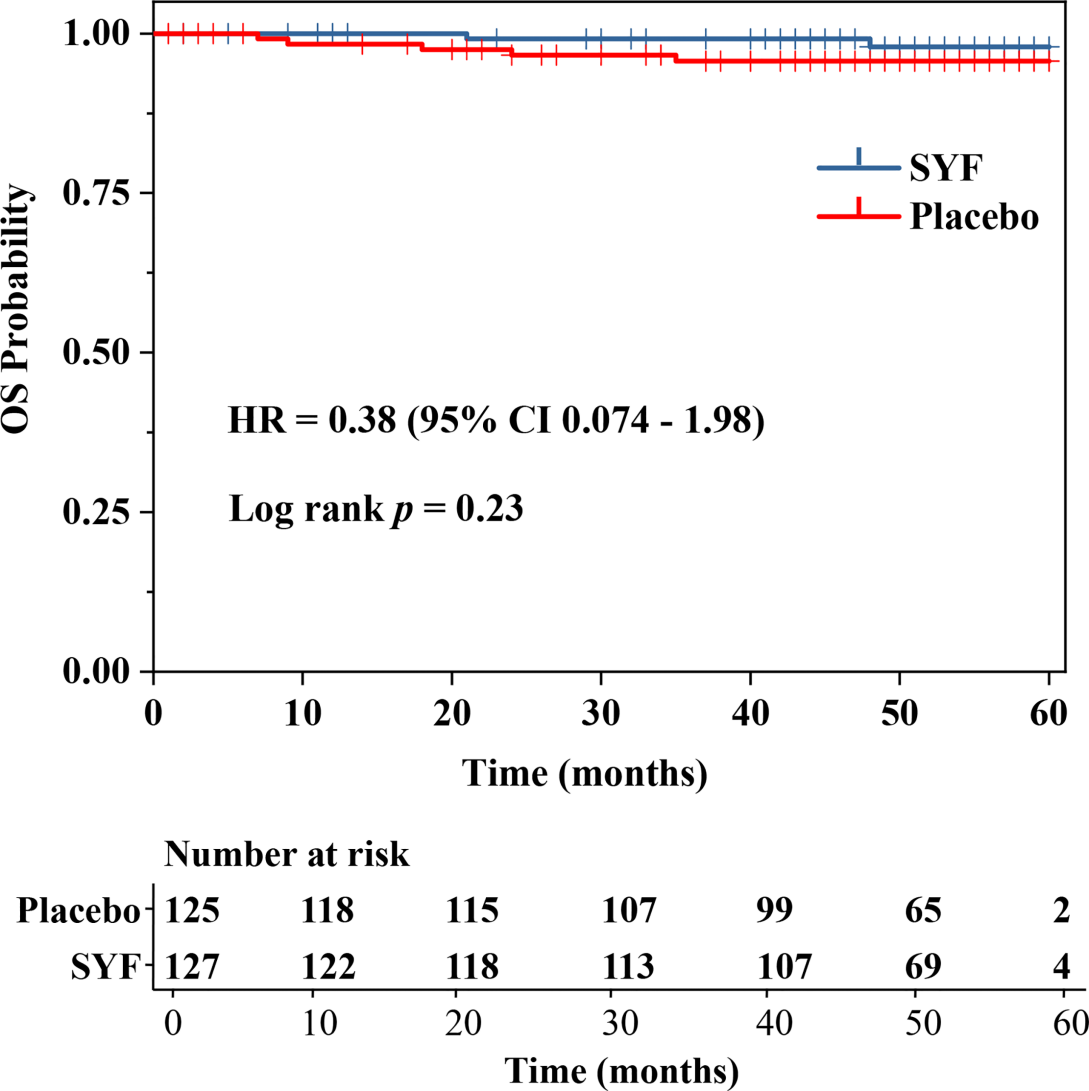
Overall Survival.

Although there was no significant difference in the Kaplan-Meier curves of OS between the two treatment groups, the number of death events in the SYF plus chemotherapy group was less than that in the placebo plus chemotherapy group (2 vs. 5, HR, 0.38; 95% CI, 0.074-1.98; stratified log-rank *P* = 0.23) (**Figure 3**). The absolute benefit for the 5-year OS rate in the SYF plus chemotherapy group was 2.2% (*P* = 0.26). However, adequate evaluation of the efficacy of SYF plus chemotherapy on OS in female patients with TNBC requires more events and long-term follow-up.

**Figure 4:**
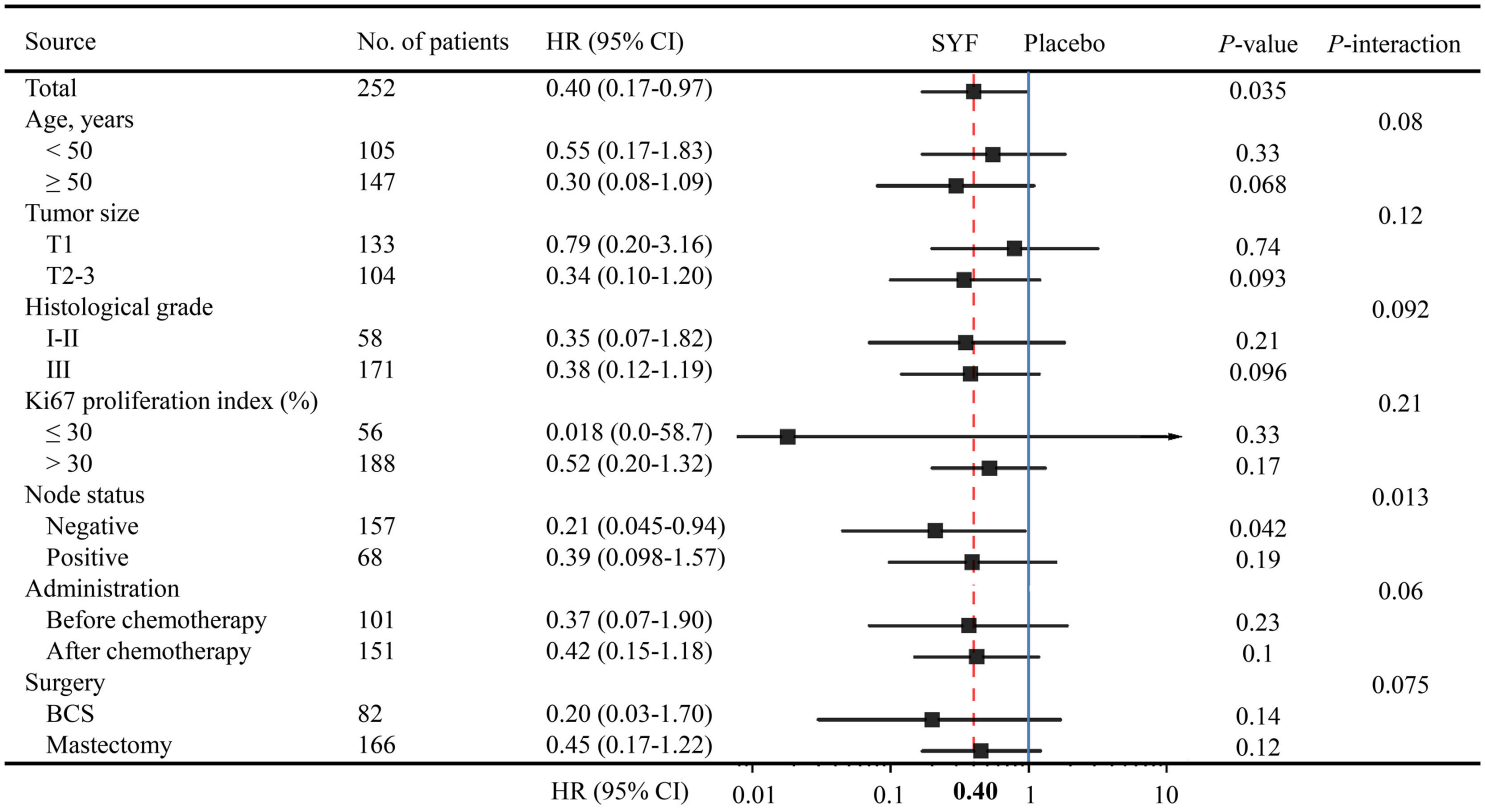
Forest plots of the exploratory subgroup analysis of DFS.

Node-negative patients benefited more from SYF plus chemotherapy treatment (HR = 0.21; 95% CI, 0.045-0.94, *P*-interaction = 0.013) in accordance with the exploratory subgroup analyses of DFS (**Figure 4**). Patients of older age, with large tumor size, and a high-grade histological appeared to have benefited more from SYF plus chemotherapy treatment (**Figure 4**). There were no marked differences among the treatment intervention times (before or after chemotherapy) or the types of surgeries (BCS and mastectomy).

### Safety

There was no significant difference in treatment-related adverse events (grades 2 to 4) between the two treatment groups throughout the trial. The two treatments were generally well tolerated. There were no treatment-related deaths or life-threatening events during the consecutive experimental observation period. Upper limb edema, pain, and diarrhea were observed in the SYF plus chemotherapy group, while liver function injury, weakness, pain, and diarrhea were observed in the placebo group. The overall treatment-related adverse event rates of the experimental group and the placebo group were 2.4% and 3.2%, respectively (**Table 2**).

## Discussion

The purpose of this RCT was to determine whether SYF has additional benefits in TNBC adjuvant therapy. The results of the present study indicated that SYF adjuvant therapy plus conventional standard chemotherapy has clear benefits compared with conventional standard chemotherapy.

According to our previous prospective investigation, SYF can increase 2-year DFS in patients with TNBC. The absolute return of 2-year DFS in the SYF plus chemotherapy group was 6.2%, and SYF plus chemotherapy reduced the rate of breast cancer recurrence and metastasis by 11% (*P* < 0.05). In the present study, the absolute return of 5-year DFS in SYF plus chemotherapy group was 8.7%. The 5-year DFS time was longer in those assigned to SYF plus conventional standard chemotherapy compared with placebo plus conventional standard chemotherapy (94.2%vs 85.5%, hazard ratio [HR] = 0.40; 95%CI, 0.17-0.97; *P* = 0.034). The results of the present RCT study not only confirmed this benefit but also further showed that SYF adjuvant therapy can maintain this benefit for a long time. The risk of metastasis and recurrence increases with the prolonged course of the patient’s disease. The survival benefit from SYF seems to increase over time. This may be realized by the inhibition of SYF in cancer cell metastasis. In subsequent subgroup analyses, TNBC patients with lymph node-negative benefit more than cases with lymph node-positive (*P*-interaction = 0.013). We will discuss this benefit in the subsequent subgroup analysis. The 5-year DFS in this trial was significantly higher than that in our previous prospective study. We believe that this may be due to two factors. One may be that the number of samples in the previous study was smaller than that in this trial, and the other may be that the tumor burden of participants in this trial was low. The present investigation involved not only a sufficient sample size but also a longer follow-up time. More importantly, this trial was a multicenter, randomized, double-blind, placebo-controlled trial that was more reliable than our previous prospective investigation. Additionally, the 5-year DFS was 94.2% in this study, which was superior to the estimated 86.5% 5-year DFS in our previous PATTERN (adjuvant platinum and taxane in triple-negative breast cancer) trial (15). The results of the present study indicated that SYF adjuvant therapy plus chemotherapy has additional significant benefits compared with recommended conventional standard chemotherapy, even compared with the current best recommended chemotherapy regimens (15). It should be noted that the patients who tend to seek traditional Chinese medicine adjuvant therapies may have a stronger desire for survival than ordinary nontraditional Chinese medicine adjuvant therapy patients (16). Thus, this potential psychological suggestion effect should not be ignored.

According to exploratory subgroup analysis, the patients who are more sensitive to the SYF combination regimen may exhibit similar characteristics, such as old age, larger tumor size, and higher grade of pathology. The subgroup analysis also showed that the benefits of the SYF regimen were significant in patients with lymph node-negative TNBC when compared to in cases with lymph node-positive (*P*-interaction = 0.013). We hypothesized that SYF can effectively inhibit the invasion and metastasis of breast cancer cells in these node-negative patients. A negative lymph node status generally suggests no clinically detectable metastasis or less metastasis, while a positive node status indicates high metastatic capability. A previous study found that lymph node metastasis is not only a marker of late diagnosis of breast cancer but also a marker of the invasive phenotype (17). Early intervention with SYF could effectively inhibit the migration, invasion, and metastasis of breast cancer cells in these patients with negative lymph node status. Although the HR of SYF was also lower (< 0.5) in lymph node-positive patients, the benefit of SYF adjuvant therapy was not significant (*P* > 0.19). There are two possible reasons for this phenomenon. The first is that SYF adjuvant therapy has no additional benefit for TNBC patients with metastases. As discussed above, a positive lymph node status usually indicates a high possibility of metastasis of cancer cells. Indeed, there is a correlation between lymph node metastasis and the tumor immune microenvironment in breast cancer (18). These lymph node-positive patients seem to develop clinically undetectable or detectable metastases. Unfortunately, SFY was ineffective in the treatment of micrometastasis of tumor cells. The second reason is that there may be too few lymph node-positive cases or too few DFS events. Due to the exaggerated 95% CI data range among the subgroups with too few samples, the phenomenon may actually be caused by the small sample size (19). Increasing the number of TNBC cases or prolonging the follow-up time may resolve this issue. In addition, we found that TNBC patients with age ≤ 35 years and regional lymph node stage N1 may be the benefit group of SYF in our previous cohort trial (13). But there were 73 and 75 cases in the exposed and non-exposed groups in our previous study, respectively (13). Therefore, although with a significant *P*-value, we believed that the results analyzed with quite a few cases were not very highly reliable. Although a larger sample size than that in our previous trial was included, a similar situation would occur if the subgroup analysis was performed according to more subgroup types in the present RCT investigation. Therefore, limited subgroup analyses were performed in this study. In the future, more direct experimental evidence should be supplied to evaluate this hypothesis. Another RCT trial with more clinical centers and more patients has been approved by the Shanghai Hospital Development Center (No. SHDC2020CR1050B). The investigation is expected to uncover these confusions, which provide more evidence and reference for the precise treatment of TNBC.

There are several limitations of this study. First, the chemotherapy regimen in this trial was not the only fixed regimen. Although epirubicin and cyclophosphamide (EC) followed by paclitaxel (EC-P) was the principal chemotherapy regimen (> 45% of the total cases) in the present study, which is recommended in the National Comprehensive Cancer Network guidelines (ECOG 1199) (20), there were also other chemotherapy regimens, such as CEF-T and AC-T (**Supplementary Table 2**). Second, the sample size of the current study was still relatively small, and the follow-up time was not long enough. Stratifying patients is difficult, especially in OS analysis. Third, due to the high heterogeneity of TNBC, it can be further subdivided into multiple subtypes (21–23). The present study did not perform a TNBC subtype analysis, and additional trials should be carried out to follow the TNBC subtypes. Identifying TNBC patients who may benefit from immunotherapy in advance, and then realizing precise immunotherapy, will be the key to improving the prognosis of TNBC patients. Based on the new TNBC classification, our recent FUTURE trial found that advanced immunomodulatory TNBC patients responded well to immunotherapy (combination regimen consisting of famitinib, camrelizumab, and nab-paclitaxel) and achieved the best objective response rate (ORR) (24). Finally, the patients who participated in this study were early-stage and operable TNBC patients. Whether the advantage of SYF is applicable to patients with advanced-stage TNBC still needs to be determined by further clinical trials.

## Conclusions

In summary, the present RDBPC trial found that compared with conventional chemotherapy regimens, the traditional Chinese medicine SYF plus conventional chemotherapy regimens may be an alternative adjuvant chemotherapy strategy for women with operable TNBC. However, high-level evidence still needs to be collected and examined before traditional Chinese medicine SYF plus chemotherapy regimens can be made the new standard of care.

## Data Availability Statement

The raw data supporting the conclusions of this article will be made available by the authors, without undue reservation.

## Ethics Statement

The studies involving human participants were reviewed and approved by Chinese Clinical Trial Registry. The patients/participants provided their written informed consent to participate in this study.

## Author Contributions

Conception and design: SL and ZS. Collection and assembly of data: CW, GL, QL, WQ, JL, ZY, YW, and SZ. Analysis and interpretation of the data: CW and CS. Drafting of the article: CW and CS. Critical revision for important intellectual content: GL, YQ, XX, XW, and QW. Provision of study materials or patients: GL, YQ, XX, XW, and QW. Obtaining of funding: SL. Final approval of the article: All authors.

## Funding

This study was supported by grants from the Shanghai Municipal Health Commission (Grant Number ZY3-LCPT-2-1002), and Science and Technology Commission of Shanghai Municipality (Grant Number 20Z21900300), as well as Shanghai Hospital Development Center (Grant Number SHDC2020CR1050B).

## Conflict of Interest

The authors declare that the research was conducted in the absence of any commercial or financial relationships that could be construed as a potential conflict of interest.

## Publisher’s Note

All claims expressed in this article are solely those of the authors and do not necessarily represent those of their affiliated organizations, or those of the publisher, the editors and the reviewers. Any product that may be evaluated in this article, or claim that may be made by its manufacturer, is not guaranteed or endorsed by the publisher.

## Supplementary Material

Supplementary Table 1 Recruitment of patients by hospital and cancer center.

Supplementary Table 2 Summary of main chemotherapy regimens. EC-P, epirubicin and cyclophosphamide plus paclitaxel (Taxol); EC, epirubicin plus cyclophosphamide; CEF-T, cyclophosphamide, epirubicin, fluorouracil and docetaxel; AC-T, adriamycin and cyclophosphamide plus paclitaxel (Taxol).

## Abbreviations

AC-T, adriamycin and cyclophosphamide plus paclitaxel (Taxol); CEF-T, cyclophosphamide, epirubicin, fluorouracil and docetaxel; ChiCTR: Chinese Clinical Trial Registry; DFS, disease-free survival; EC, epirubicin plus cyclophosphamide; EC-P, epirubicin and cyclophosphamide plus paclitaxel (Taxol); ER, estrogen receptor; HER2, human epidermal growth factor receptor 2; ORR, objective response rate; OS: overall survival; PR: progesterone receptor; RCT, randomized controlled trial; RDBPC, randomized, double-blind, placebo-controlled trial; SYF, Sanyin formula; TCM, traditional Chinese medicine; TNBC, triple-negative breast cancer.
