# Supplementary material for: Effectiveness of the Sanyin Formula Plus Chemotherapy on Survival in Women With Triple-Negative Breast Cancer: A Randomized Controlled Trial
Source: Front Oncol. 2022 May 26;12:850155. doi: 10.3389/fonc.2022.850155 (PMC9197261; doi:10.3389/fonc.2022.850155)
Supplement: Supplementary Table 1 — Recruitment of patients by hospital and cancer center. [file Table_1.docx]

**Table S1. Recruitment of patients by hospital and cancer center.**

| Hospital and cancer center | N = 252 |
| --- | --- |
| Longhua Hospital, Shanghai University of Traditional Chinese Medicine | 106 |
| Shanghai Cancer Center, Fudan University | 73 |
| Yueyang Hospital of Integrated Traditional Chinese and Western Medicine, Shanghai University of Traditional Chinese Medicine | 30 |
| Shuguang Hospital, Shanghai University of Traditional Chinese Medicine | 28 |
| Shanghai Traditional Chinese Medicine Hospital, Shanghai University of Traditional Chinese Medicine | 15 |
