# Supplementary material for: Effectiveness of the Sanyin Formula Plus Chemotherapy on Survival in Women With Triple-Negative Breast Cancer: A Randomized Controlled Trial
Source: Front Oncol. 2022 May 26;12:850155. doi: 10.3389/fonc.2022.850155 (PMC9197261; doi:10.3389/fonc.2022.850155)
Supplement: Supplementary Table 2 — Summary of main chemotherapy regimens. EC-P, epirubicin and cyclophosphamide plus paclitaxel (Taxol); EC, epirubicin plus cyclophosphamide; CEF-T, cyclophosphamide, epirubicin, fluorouracil and docetaxel; AC-T, adriamycin and cyclophosphamide plus paclitaxel (Taxol). [file Table_2.docx]

**Table S2. Summary of main chemotherapy regimens.**

| Types | No. (%) of patients | |
| --- | --- | --- |
|  | Placebo (N = 125) | SYF (N = 127) |
| EC-P | 57 (45.6) | 61 (48.0) |
| EC | 15 (12.0) | 18 (14.2) |
| CEF-T | 14 (11.2) | 12 (9.4) |
| AC-T | 7 (5.6) | 5 (3.9) |
| Others | 32 (25.6) | 31 (24.8) |

EC-P, epirubicin and cyclophosphamide plus paclitaxel (Taxol); EC, epirubicin plus cyclophosphamide; CEF-T, cyclophosphamide, epirubicin, fluorouracil and docetaxel; AC-T, adriamycin and cyclophosphamide plus paclitaxel (Taxol).
